# Assertive outreach treatment versus care as usual for the treatment of high-need, high-cost alcohol related frequent attenders: study protocol for a randomised controlled trial

**DOI:** 10.1186/s12889-020-8437-y

**Published:** 2020-03-14

**Authors:** R. Blackwood, A. Wolstenholme, A. Kimergård, S. Fincham-Campbell, Z. Khadjesari, S. Coulton, S. Byford, P. Deluca, S. Jennings, E. Currell, J. Dunne, J. O’Toole, J. Winnington, E. Finch, C. Drummond

**Affiliations:** 1 National Addiction Centre, Institute of Psychiatry, Psychology and Neuroscience, King’s College London, London, UK; 2 University of Kent, Kent, Canterbury, UK; 3 King’s Health Economics, Institute of Psychiatry, Psychology & Neuroscience at King’s College London, London, UK; 4 NIHR Collaborations for Leadership in Applied Health Research and Care South London, London, UK; 5 South London and the Maudsley NHS Foundation Trust, London, UK

**Keywords:** Alcohol, Dependence, Frequent attenders, Assertive outreach treatment, Randomized controlled trial, Multimorbidity, High need high cost

## Abstract

**Background:**

Alcohol-related hospital admissions have doubled in the last ten years to > 1.2 m per year in England. High-need, high-cost (HNHC) alcohol-related frequent attenders (ARFA) are a relatively small subgroup of patients, having multiple admissions or attendances from alcohol during a short time period. This trial aims to test the effectiveness of an assertive outreach treatment (AOT) approach in improving clinical outcomes for ARFA, and reducing resource use in the acute setting.

**Methods:**

One hundred and sixty ARFA patients will be recruited and following baseline assessment, randomly assigned to AOT plus care as usual (CAU) or CAU alone in equal numbers. Baseline assessment includes alcohol consumption and related problems, physical and mental health comorbidity and health and social care service use in the previous 6 months using standard validated tools, plus a measure of resource use. Follow-up assessments at 6 and 12 months after randomization includes the same tools as baseline plus standard measure of patient satisfaction. Outcomes for CAU + AOT and CAU at 6 and 12 months will be compared, controlling for pre-specified baseline measures. Primary outcome will be percentage of days abstinent at 12 months. Secondary outcomes include emergency department (ED) attendance, number and length of hospital admissions, alcohol consumption, alcohol-related problems, other health service use, mental and physical comorbidity 6 and 12 months post intervention. Health economic analysis will estimate the economic impact of AOT from health, social care and societal perspectives and explore cost-effectiveness in terms of quality adjusted life years and alcohol consumption at 12-month follow-up.

**Discussion:**

AOT models piloted with alcohol dependent patients have demonstrated significant reductions in alcohol consumption and use of unplanned National Health Service (NHS) care, with increased engagement with alcohol treatment services, compared with patients receiving CAU. While AOT interventions are costlier per case than current standard care in the UK, the rationale for targeting HNHC ARFAs is because of their disproportionate contribution to overall alcohol burden on the NHS. No previous studies have evaluated the clinical and cost-effectiveness of AOT for HNHC ARFAs: this randomized controlled trial (RCT) targeting ARFAs across five South London NHS Trusts is the first.

**Trial registration:**

International standard randomized controlled trial number (ISRCTN) registry: ISRCTN67000214, retrospectively registered 26/11/2016.

## Background

Worldwide, alcohol use was the seventh leading risk factor for both deaths and DALYs (Disability-Adjusted Life year) in 2016, accounting for 2.2% of female deaths and 6.8% of male deaths. In 2016, alcohol use led to 2.8 million deaths worldwide and was the leading risk factor for premature death and disability among people aged 15–49 years. Alcohol use accounts for nearly 10% of global deaths among populations aged 15–49 years [1].

In the UK, alcohol use disorders are a major public health challenge: it is the leading cause of preventable disability in working age men [2, 3] and the third greatest risk factor for years lived with disability [4]. Deaths from liver disease have increased by over 400% since the 1970s in the UK [5], and predicted to overtake cardiovascular disease as the leading cause of death by 2021 [6].

The UK Department of Health estimates that alcohol costs the NHS in England £3.5bn per annum, 80% of which is incurred in inpatient and emergency care costs [7]. However, the wider annual costs to society in England are estimated to be considerably greater at £21bn, £11bn of which is incurred by the criminal justice system [8].

Alcohol-related hospital admissions have doubled in the last 10 years to over 1.1 m per annum in England, a quarter (337,000) of which were for conditions wholly attributable to alcohol [9]. Reducing alcohol related admissions is a key public health target and is an indicator in the Public Health Outcomes Framework [10]. High-need, high-cost (HNHC) alcohol related frequent attenders (ARFAs) are a subgroup of all patients with alcohol-related hospital admissions and are characterised by having multiple hospital admissions or attendances caused by alcohol^1^ during a relatively short time period. Whilst there is no single universal definition for an ARFA, for the purposes of this trial we defined an ARFA as someone who has had two or more admissions in any of the participating NHS trusts for at least one wholly attributable alcohol diagnoses within a 1 year period, OR; has had at least ten presentations to an emergency department in any of the participating NHS trusts within a 1 year period, OR; has at least four presentations to an emergency department in any of the participating NHS trusts within a month, OR; has been admitted at least once for a wholly attributable alcohol diagnosis and had at least four presentations to an emergency department in any of the participating NHS trusts within a 1 year period.

Previous studies show that the HNHC ARFA patient group typically have high levels of multimorbidity, including both physical and mental illness, social isolation, poor quality of life, unstable housing or homelessness, high criminal justice involvement, unemployment and living on benefits [11]. Notably, they either have poor or no engagement with existing specialist alcohol treatment services. Their Accident and Emergency Department (A&E) attendance is frequent, often due to not being registered with a General Practitioner (GP), and they have frequent emergency hospital admissions to both acute and mental health beds. Qualitative research has also identified that as a group ARFAs experience high levels of stigma and social exclusion [12].

An intensive assertive outreach treatment (AOT) intervention has been developed to target the patients who have the highest alcohol related hospital attendance. AOT is based on a model of community service provision originally designed for people with severe mental illness (SMI) and was first pioneered by the work of Stein and Test (1980) [13]. AOT emphasises active engagement over an extended period [14, 15] and has several features which distinguish it from usual care, including: (i) rapid access to services, (ii) a small caseload, (iii) a high ratio of community to office-based appointments, (iv) assertive engagement (e.g. with multiple attempts) and (v) a shared care approach, with care coordinators working within a multidisciplinary team that meets frequently [16, 17]. In adult mental health care, AOT (sometimes also referred to as Assertive Community Treatment) is an extensively researched and widely used model of care for treating difficult to engage patients with severe and enduring mental illness. AOT and its elements have also been used in the treatment of alcohol dependence [18, 19] which clinically, like severe and enduring mental illness, also often presents as a chronic relapsing disorder with high public health costs [20–25]. An AOT model implemented with alcohol dependent patients, demonstrated significant reductions in alcohol consumption and use of unplanned NHS care, as well as increased engagement with alcohol treatment services, compared with patients receiving care as usual [14]. A national survey of AOT services for ARFAs in 2017 [21] found 76 services across England offering AOT for ARFAs between December 2015 and June 2016. The majority included a multi-disciplinary team and offered extended support including advice on housing, mental and physical health in addition to alcohol treatment, however there was considerable variation amongst the models of care delivered and no previous RCTs have evaluated their clinical effectiveness and cost-effectiveness.

While AOT interventions are more costly per case than current standard care in the UK, the rationale for targeting HNHC ARFAs is that they incur a disproportionately high amount of the overall alcohol burden on the NHS. Hence the potential savings to the NHS, as well as to wider social and criminal justice services, from a successful intervention outcome are greater than other less intensive interventions for less complex patients.

## Methods

### Aim

This randomised controlled trial will assess the clinical and cost-effectiveness of care as usual (CAU) plus AOT compared with CAU alone for adults who have frequent alcohol related admissions.

### Objectives

1. To conduct a randomised controlled trial of CAU + AOT versus CAU in a sample of alcohol related frequent attenders in five NHS trusts in South East London.
2. To evaluate the effectiveness and cost-effectiveness of CAU + AOT versus CAU alone in ARFAs to acute and mental health inpatient care.
3. To describe the characteristics and health and social care needs of ARFAs.
4. To identify potential mechanisms of action of AOT and identify patient sub-groups for whom AOT is more effective.

### Primary hypothesis

- CAU + AOT will be more effective than CAU alone in increasing the proportion of days abstinent from alcohol, measured by the Time Line Follow Back (TLFB) form 90, at 12 months follow-up.

### Secondary hypotheses

- CAU + AOT will be cost-effective compared with CAU alone at 12 months follow-up.
- CAU + AOT will reduce unplanned health care utilisation including admissions to Emergency Departments (ED) and acute and mental health inpatient care compared to CAU alone.
- CAU + AOT will increase engagement with specialist addiction and recovery services compared to CAU alone.
- Addiction treatment engagement, readiness to change and therapeutic alliance will moderate treatment effectiveness.

### Assertive outreach treatment intervention

Patients in the intervention arm of the study will receive AOT in addition to usual care. AOT will incorporate the following elements [14]:

(i) Each AOT practitioner should have a maximum caseload of 15 AOT patients.
(ii) Care provided by a multidisciplinary team (including psychiatrists, community addiction nurses, substance use disorder specialists, community support workers).
(iii) Frequent and regular contact between practitioner and patient (at least once a week predominantly in patients’ homes and local community settings).
(iv) Assertive engagement – persistent and repeated attempts by the practitioner to make contact, with the emphasis being on maintaining contact and building relationships.
(v) Content of sessions will focus on both health and social care needs – including accommodation, leisure, occupation, and physical and mental health, with an emphasis on a patient-led agenda.
(vi) Flexibility – practitioners should work flexibly with patients’ goals and include those which are less related to the addiction. The AOT model has abstinence from alcohol as its ultimate clinical goal. However, whilst all treatment aspires to achieve full recovery, it can be an unrealistic short-term goal for more severely dependent drinkers with complex social support needs plus multiple comorbidities, in which case a reduction in harmful drinking will be a more realistic goal.
(vii) Openness – practitioners explain their role and aims in care planning and in visits.
(viii) Practitioners operate more broadly than in their usual professional roles to provide a wide range of support to patients.
(ix) Extended care – AOT will be provided for a period of 1 year during which patients will be introduced to other existing community services who will ultimately provide them with on-going support in the longer-term.

AOT will be used to proactively engage with ARFA patients, to assess their physical and mental health and wider social needs, then to develop shared treatment goals, and to facilitate and coordinate engagement with the relevant care and support to address their multiple presenting needs. The AOT will mainly take place either in the patient’s home or local community settings. A treatment manual has been developed by the research team as part of a previous clinical trial [11].

### Training

All staff who will be delivering the AOT service will participate in a training programme, focusing on:

- Key features of AOT and how it should be delivered in the context of alcohol-related frequent attenders, including techniques to maximise engagement.
- Overview of the research trial, inclusion and exclusion criteria, obtaining consent, the assessment tools being used in the trial, maintaining contact logs.
- Safety whilst working in the community.

In addition, each member of staff will spend a day shadowing mental health assertive outreach staff participating in community and home visits with patients. This will also provide staff with the opportunity to ask questions around practical issues associated with delivering AOT.

### Control intervention

All participants in the control arm of the study will receive CAU i.e. routine clinical practice, so that the hypothesis that CAU + AOT is more effective than CAU alone can be tested. CAU typically involves treatment of presenting physical or mental health problems and referral to specialist addiction services for ongoing help with alcohol related problems. Patients receiving CAU have access to medical detoxification, psychological interventions targeting drinking behaviour and aftercare, which are usually provided by specialist addiction services. But in contrast to AOT, the emphasis will be on the patient’s own motivation to engage with available services. Specialist input from addictions psychiatry, clinical psychology and social work may also be a part of CAU but the assertive and regular nature of contacts in AOT does not feature.

### Intervention fidelity

Prior to delivering AOT all staff will undergo training as outlined above. Practitioners will attend regular weekly multidisciplinary clinical meetings and regular individual and group supervision to ensure that the intervention is being delivered in a consistent manner by all practitioners, in line with the characteristic features of AOT. Staff providing care to patients will be asked to complete a log of the contacts they have with patients and the care provided. This will include the nature of the contact (face-to-face meetings, telephone calls, text messages and emails), setting (home, community, clinical), the focus of the contact (mental health, physical health, housing, finance etc), duration of the contact, and the member of staff involved.

### Participants

#### Inclusion criteria

i) Age 18 years or over.
ii) Able to understand English sufficiently well to obtain informed consent and complete the assessment instruments.
iii) has had two or more admissions in any of the participating NHS trusts for at least one wholly attributable alcohol diagnoses within a 1 year period, OR; has had at least ten presentations to an emergency department in any of the participating NHS trusts within a 1 year period, OR; has had at least four presentations to an emergency department in any of the participating NHS trusts within a month, OR; has been admitted at least once for a wholly attributable alcohol diagnosis and had at least four presentations to an emergency department in any of the participating NHS trusts within a 1 year period.
iv) International Classification of Diseases version 10 (ICD-10) diagnosis of alcohol dependence.
v) Willing to provide informed consent to participate in the trial.
vi) A resident of Lambeth and Southwark and admitted to one of the five participating NHS Trusts.

#### Exclusion criteria

i) Unable to give informed consent.
ii) Experiencing severe mental of physical illness likely to preclude active participation in the treatment or research follow up.
iii) Already in receipt of assertive outreach services or participation in another trial.
iv) Has a severe cognitive impairment as determined by Mini Mental State Examination score of ≤10 [22].
v) Has a history of violence to staff or is registered under multi-agency public protection arrangements (MAPPA).
vi) Dependence on opiates or stimulants.
vii) Street homeless or has no recourse to public funds.

### Identification and recruitment of potential participants

The AOT clinical team will work with informatics teams in the five NHS trusts (King’s College Hospital (KCH)-, Guy’s and St Thomas’ (GST)-, South London and Maudsley (SLaM)- and St George’s Healthcare (STG)- NHS Foundation Trusts; and Lewisham and Greenwich (LGT) NHS Trust) to identify HNHC ARFA based on primary or secondary clinical diagnoses of alcohol related admissions and number of admissions or emergency department attendances in the previous 12 months. This will also include the use of the clinical research information system (CRIS) within SLaM. The clinical team will also have access to electronic clinical records and discuss potentially eligible patients with clinicians in inpatient units in the five trusts, and the Alcohol Care Teams at KCH, STG and GST, to identify additional patients who meet the study criteria but are not captured by diagnostic criteria in electronic records.

Once potentially eligible patients have been identified, they will be contacted by the AOT clinical team and assessed for eligibility and informed about the potential to participate in the trial. They will be given the Patient Information Sheet and the trial will be explained verbally. Patients will be encouraged to ask questions or consult carers, relatives or their GP. If willing to proceed, eligible patients will be introduced to a member of the research team who will again explain the trial, confirm the patient’s eligibility, and obtain informed consent. The minimum period of time between informing the patients of the trial and obtaining consent will be 24 h. Patients unwilling to take part will be offered the standard CAU, including referral to local specialist addiction services.

Patients who provide informed consent will undergo a baseline research assessment conducted by the research team and will be randomised using an electronic trial management tool. In order to maintain blinding of researchers, the treatment allocation will be provided only to the AOT clinical team who will then initiate AOT treatment or referral to specialist addictions services as appropriate.

### Baseline assessment

(i) Socio-demographic information: age, sex, ethnicity, marital status, living arrangement, children, living arrangements of children, education.
(ii) Drinking in the previous 90 days – TLFB 90I [23]. The TLFB is a validated interview method to measure alcohol consumption in the previous 90 days and is the gold standard outcome measure in alcohol treatment research. Percentage days abstinent, units of alcohol per drinking day (1 UK unit = 8 g alcohol), time to first alcoholic drink, relapse to any drinking and relapse to heavy drinking (8+/6+ units for males/females on a single occasion) will be computed.
(iii) History of drug use – TLFB.
(iv) Alcohol related problems - Alcohol Problems Questionnaire (APQ) [24]. The APQ is a validated 46-item questionnaire assessing potential problems with psychological, physical, social, legal, employment, relationships and parenting that may be experienced due to alcohol.
(v) Severity of alcohol dependence - Severity of Alcohol Dependence Questionnaire (SADQ) [25]. The SADQ is a validated 20-item self-complete questionnaire containing items representing five domains of the alcohol dependence syndrome: (i) physical withdrawal signs (ii) psychological withdrawal signs (iii) withdrawal relief drinking (iv) tolerance (v) reinstatement following a period of abstinence.
(vi) Health related quality of life – EuroQol 5 dimensions 5 levels (EQ-5D-5 L) [26]. The EQ-5D-5 L consists of five dimensions (mobility, self-care, usual activities, pain/discomfort and anxiety/depression) scored on five levels (no problems, slight problems, moderate problems, severe problems and extreme problems). The EQ-5D-5 L is an extension of the original 3-level EQ-5D and has been shown to be valid [27].

This version of EuroQol measure of health-related quality of life capable of generating quality adjusted life years (QALYs) for use in economic evaluations.

(vii) Health Related Quality of life – Short-form-12 (SF-12) [28]-a 12 item subset of Short form-36, for measuring self-reported health related quality of life.
(viii) Motivation to change - Readiness to Change - Treatment version [29]- a 12 item questionnaire used to assess the subject’s stage of change.
(ix) Social network involvement - Important People and Activities Inventory [30]. This measure gathers information regarding general and alcohol-specific types of support. This scale contains 11 indices which in combination create a summary measure for predicting alcohol use,
(x) Resource use – Adult Service Use Schedule (AD-SUS) [31] The AD-SUS is a resource use measure designed for and applied to mental health populations, including drug and alcohol populations.

### Follow up assessments

**Fig. 1:**
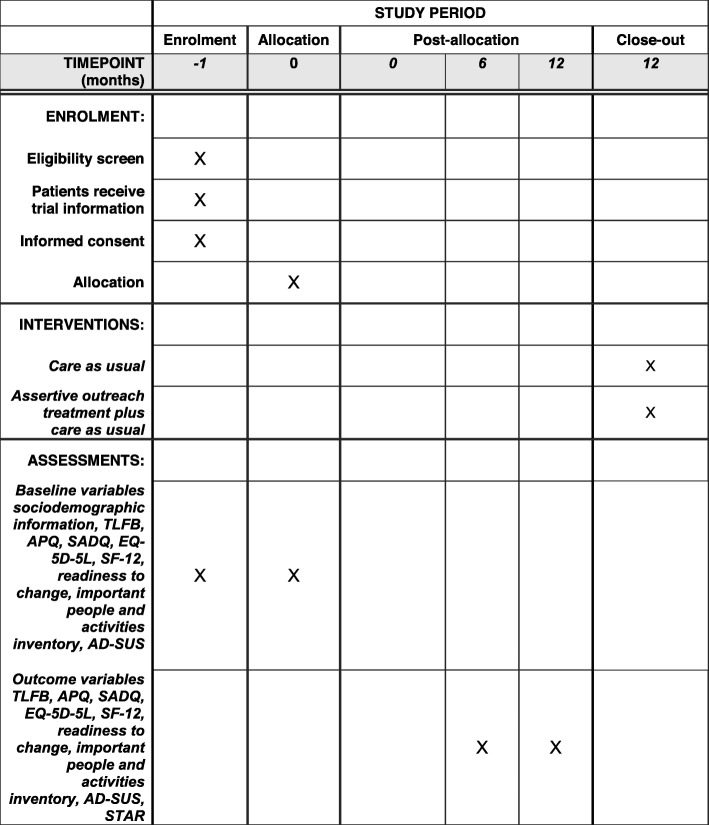
SPIRIT diagram for the schedule of enrolment, interventions, and assessments

Six and twelve months after randomisation, all participants will be followed up (see Fig. 1). A research worker, blind to treatment allocation, will arrange a convenient time to meet with the participant. Baseline measures (items i-x above) will be repeated in addition to:

(xi) Therapeutic relationship – Scale to assess therapeutic relationship (STAR) [32] completed at 12 months follow-up. Therapeutic relationship (or alliance) has been found to predict clinical outcome across a range of mental disorders including alcohol dependence. This will be used as an additional process measure to assess the impact of therapeutic relationship on medication adherence and clinical outcome.

Maintaining the researcher’s blindness to treatment allocation is an acknowledged problem in psychosocial intervention trials in comparison to placebo controlled medication trials. In order to reduce the risk of participants inadvertently revealing the treatment allocation, researchers will advise participants not to discuss their ongoing treatment arrangements or the identity of their key worker or service and will conduct the TLFB measure before discussing service utilisation as in a previous trial [11].

### Randomisation and blinding

Randomisation to one of the two treatment arms will take place after consent and baseline data have been collected, by a remote electronic allocation system. Once baseline data has been submitted on the electronic device by the researcher, the system will email the randomised allocation to a member of the clinical team. The researchers will be blind to the allocated intervention throughout the trial. The online system will be developed and maintained by Codeface Ltd. Randomisation will be stratified by site and severity of alcohol dependence (< = 30 and > 30), as measured by the Severity of Alcohol Dependence Questionnaire (SADQ).

### Sample size and power

The primary outcome of the RCT is percent days abstinent using validated Time-Line Follow-Back (TLFB) interview method over a 90-day reference period at 12 months post-recruitment, which is the gold standard in alcohol treatment research. Similar studies have used effect sizes of 0.3–0.5 in their sample size calculations [14]. We propose using an effect size of 0.4, alongside type 1 error rate, α = 0.05 and type 2 error rate, β = 0.8 and using a two-sided test. This indicates a sample size of 64 patients each in intervention and control group included in the analysis at the primary end-point. In order to address potential loss to follow-up we have incorporated a loss of 20%, larger than previous studies such as ACTAD that had a loss to follow-up of 7% at 12 months but smaller than UKATT [33] that has a 28% loss at 12 months. This inflates the required sample size at recruitment to 80 in each group a total of 160 at baseline with the expectation that at least 64 in each group, a total of 128, will be followed up at 12 months.

### Statistical analysis

Our analysis of the primary outcome measure involves a comparison of the effectiveness of AOT + CAU versus CAU alone. Analysis will be conducted by intention to treat principles and the primary outcome measure will be percent days abstinent in the 90 days prior to 12 months follow up. AOT + CAU will be compared to CAU using analysis of covariance including stratification variables; SADQ and site as covariates. Multiple imputation and sensitivity analysis will be employed to assess the impact of missing data. Results will be presented as mean differences with associated 95% confidence intervals. Secondary analysis will address drinking and other outcomes at 6 and 12 months using appropriate modelling approaches and these will be adjusted for covariates; SADQ, site, age and gender. We will explore the association between engagement in treatment (measured by cumulative number of days in AOT and specialist alcohol treatment), readiness to change, and therapeutic alliance, and drinking outcomes using a linear regression adjusted for known confounders. As with most trials the analysis plan will be refined throughout the course of the study and the final analysis plan prepared and agreed by the research team and Trial Steering Committee.

### Economic evaluation

The economic evaluation will estimate the costs and cost-effectiveness of CAU + AOT compared with CAU alone over the 12-month follow-up. The primary economic evaluation will take a societal perspective including the use of all health and social care services, plus productivity losses (time off work due to illness) and criminal justice sector resources, known to be of particular importance in drug and alcohol populations. Whilst the NHS/personal social services perspective is generally preferred by National Institute for Health and Care Excellence (NICE), the inclusion of criminal justice sector resources is recognised by NICE as an appropriate extension for drug and alcohol treatment evaluations [34].

Resource use data is being measured using a version of the AD-SUS which was developed for addiction and alcohol use disorder populations in previous research [31]. The AD-SUS is completed in interview with participants at baseline (covering the previous 6 months) and at 6 and 12-month follow-up (covering the period since previous interview). Appropriate unit costs will be applied to all resource elements reported by participants to calculate the total cost per participant. The AOT intervention will be costed directly, taking a micro-costing approach and nationally applicable unit costs will be applied to all other health and social care services used (for example, NHS Trust reference costs and the Personal Social Services Research Unit Costs of Health and Social Care compendium [35]. Criminal activity will be costed using existing Home Office estimates [36], uprated as appropriate, unless newer estimates become available. Productivity losses will be valued using the human capital approach, which involves multiplying time off work due to illness by an appropriate wage rate [37].

Health utility is being measured with the EQ-5D-5 L (five dimensions, five levels) version of the EuroQol measure of health-related quality of life. The EQ-5D is a measure capable of generating quality adjusted life years (QALYs) for use in economic evaluations and will be used in the primary economic evaluation exploring cost-effectiveness in terms of cost per QALY. Responses will generate health states that utility weights can then be applied to and QALYs will be calculated using the total area under the curve approach. EQ-5D-5 L is a more sensitive version of the EQ-5D, measuring health-related quality of life and has been used extensively in previous alcohol studies in the UK (e.g. UKATT Research Team [33]).

Two economic evaluations will be carried out: a cost-utility analysis using quality adjusted life years calculated from the EQ-5D-5 L [38] using the area under the curve approach [39] (primary) and a cost-effectiveness analysis using the primary clinical measure of the RCT, percentage days abstinent in the past 90 days using the Time Line Follow Back interview (secondary). Cost-effectiveness will be assessed through the calculation of incremental cost-effectiveness ratios (ICER) – the additional costs of one intervention compared to another divided by the additional effects of one intervention compared to another [40]. Uncertainty will be represented by cost-effectiveness acceptability curves [41]. Analyses will use standard parametric tests with mean differences and 95% confidence intervals generated from bootstrap regressions (1000 replications) to account for non-normal distribution common to economic data [42, 43]. In line with the clinical analyses, all economic analyses will be conducted by intention to treat principles with stratification variables, SADQ and site as covariates and multiple imputation by chained equations to account for missing data. Sensitivity analysis will explore the impact of missing data using a complete case approach and the impact of perspective taking the narrower NHS/personal social services.

### Protocol amendments

The inclusion criteria changed during recruitment to also include patients with alcohol related frequent emergency department attendances.

## Discussion

AOT models piloted with alcohol dependent patients have demonstrated significant reductions in alcohol consumption and use of unplanned NHS care, with increased engagement with alcohol treatment services, compared with patients receiving CAU. While AOT interventions are more costly per case than current standard care in the UK, the rationale for targeting HNHC ARFA is because of their disproportionate contribution to the overall alcohol burden on the NHS. No previous studies have evaluated the clinical and cost-effectiveness of AOT for HNHC ARFA: this RCT targeting ARFA across five South London NHS Trusts is the first to do so.

The inclusion criteria changed during recruitment to also include patients with alcohol related frequent emergency department attendances. In practice, this resulted in an increase in recruitment across the participating NHS trusts. Assessments completed by the AOT clinical team during the first 6 months of recruitment revealed that some participants had few inpatient hospital admissions in a year, but frequently attended an emergency department for alcohol related reasons, often choosing to leave before discharge. A high number of emergency department attendances with no resulting inpatient hospital admission incur costs of similar proportions to an inpatient hospital admission which are of importance to the economic analysis undertaken in the trial. A full amendment was approved to recruit from this patient population.

As far as we are aware, this is the first randomised controlled trial to evaluate AOT approaches for high cost high need alcohol related frequent attenders. The main strength of the study is that participants are randomized to receive the intervention or care as usual (control). By randomly allocating ARFA to control or intervention groups the impact of confounders (such as age, gender, ethnicity, levels of engagement with other services) as a source of bias are equally distributed between the intervention and control groups, and therefore minimised. The blinding of the researchers to treatment allocations reduces reporting bias. The economic analysis is carried out to include criminal justice sector resources which is recognised as an appropriate extension for drug and alcohol treatment evaluations.

Limitations include the potential of loss of subjects to follow-up. To reduce this risk, most data collection takes place in the subject’s home or community environment. In this hard to reach cohort of interest, if loss to follow-up does occur, using multiple imputation to account for missing data will help minimize this impact. Another potential challenge is maintaining the researcher’s blindness to treatment allocation, which is an acknowledged problem in psychosocial intervention trials. Researchers will advise participants not to discuss their ongoing treatment arrangements or the identity of their key worker or service and will conduct the TLFB measure before discussing service utilisation. The clinical complexity of the HNHC ARFA means that many experience mental health problems, homelessness and drug addiction and a limitation of our study is that by the nature of our exclusion criteria, we are not testing the efficacy of AOT on certain subgroups of the HNHC ARFA population. Consideration will need to be given as to whether the full extent of clinical and economic benefits have been completely realised within the 12 month follow up period; and how rates of death in both groups and signposting to health services impact on overall costs.

### Trial status

Analysis is ongoing.

## Data Availability

This manuscript does not contain any data. At the end of the study arrangements will be made to make available anonymised datasets through a public repository.

## Abbreviations

A&E: Accident and Emergency Department
ACTAD: Assertive community treatment for alcohol dependence
AD-SUS: Adult service use schedule
AOT: Assertive outreach treatment
APQ: Alcohol problem questionnaire
ARFA: Alcohol related frequent attender
CAU: Care as usual
CRIS: Clinical Research Information System
DALY: Disability adjusted life year
ED: Emergency department
EQ-5D-5 L: Euorqol 5 dimension 5 level
GP: General Practitioner
GST: Guy’s and St Thomas’ NHS Foundation Trust
HNHC: High need high cost
ICD-10: International Classification of Diseases, version 10
ICER: Incremental cost effectiveness ratio
ISRCTN: International standard randomized controlled trial number
KCH: King’s College Hospital NHS Trust
LGT: Lewisham and Greenwich NHS Trust
MAPPA: Multi-agency Public Protection Arrangements
NHS: National Health Service
NICE: National Institute for Health and Care Excellence
QALY: Quality adjusted life year
RCT: Randomized controlled trial
SADQ: Severe alcohol dependence questionnaire
SF-12: Short form 12
SLaM: South London and the Maudsley NHS Foundation Trust
SMI: Severe mental illness
STAR: Scale to assess therapeutic relationship
STG: St George’s NHS Foundation Trust
TLFB: Timeline follow back

## Footnotes

1 Caused by alcohol means that one of the given ICD10 diagnoses in hospital records can be wholly attributable to alcohol.
